# Recent Advances in Organ‐on‐Chips Integrated with Bioprinting Technologies for Drug Screening

**DOI:** 10.1002/adhm.202203172

**Published:** 2023-05-14

**Authors:** Nima Tabatabaei Rezaei, Hitendra Kumar, Hongqun Liu, Samuel S. Lee, Simon S. Park, Keekyoung Kim

**Affiliations:** ^1^ Department of Mechanical and Manufacturing Engineering University of Calgary Calgary Alberta T2N 1N4 Canada; ^2^ Department of Pathology and Laboratory Medicine Cumming School of Medicine University of Calgary Calgary Alberta T2N 1N4 Canada; ^3^ Liver Unit Cumming School of Medicine University of Calgary Calgary Alberta T2N 1N4 Canada; ^4^ Department of Biomedical Engineering University of Calgary Calgary Alberta T2N 1N4 Canada

**Keywords:** 3D bioprinting, biofabrication, drug screening, microfluidics, organ‐on‐chips (OOCs)

## Abstract

Currently, the demand for more reliable drug screening devices has made scientists and researchers develop novel potential approaches to offer an alternative to animal studies. Organ‐on‐chips are newly emerged platforms for drug screening and disease metabolism investigation. These microfluidic devices attempt to recapitulate the physiological and biological properties of different organs and tissues using human‐derived cells. Recently, the synergistic combination of additive manufacturing and microfluidics has shown a promising impact on improving a wide array of biological models. In this review, different methods are classified using bioprinting to achieve the relevant biomimetic models in organ‐on‐chips, boosting the efficiency of these devices to produce more reliable data for drug investigations. In addition to the tissue models, the influence of additive manufacturing on microfluidic chip fabrication is discussed, and their biomedical applications are reviewed.

## Graphical abstract

Demands for reliable drug screening and alternatives to animal studies, organ‐on‐a‐chips, are attracting researchers’ attention as one of the most promising biomedical tools for this mean. This manuscript contains a summary of different bioprinting techniques to facilitate the construction of more complex and relevant biological models, enhancing automation and reproducibility and making the process high throughput.

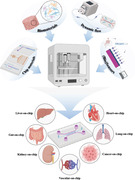

## 1 Introduction

Despite significant improvements in diagnosis, treatment, and prevention of various diseases, the emergence of new diseases poses an urgent global challenge.^[^ ^1^ ^]^ Newly emerging diseases remain a critical parameter in human health and society. As an example, one of the most important that we are now facing, COVID‐19, has caused tremendous mortality. Additionally, there are several more fatal diseases which cannot be treated currently due to a lack of knowledge about their mechanisms, as well as difficulty in drug development. With the increasing cost of clinical trials, pharmaceutical and biotechnology companies are less willing to spend millions of dollars to undertake clinical investigations,^[^ ^2^ ^]^ moreover, drug testing process is extensively time consuming. This is in large part because approximately 40% of newly developed drugs, even after accomplishing preclinical evaluation with animal models, fail clinical trials.^[^ ^3^ ^]^ Conventionally, due to its simplicity, scalability and reproducibility, 2D cell culture has been used for drug evaluation.^[^ ^4^ ^]^

Nevertheless, when comparing 2D and 3D models, there are significant differences, especially in terms of proteins, biomarkers, gene expression,^[^ ^5^, ^6^, ^7^ ^]^ drug response, resistance,^[^ ^6^, ^7^ ^]^ cell morphology,^[^ ^8^, ^9^ ^]^ and cell growth and migration.^[^ ^10^, ^11^ ^]^ By utilizing 3D models, cell–cell and cell–matrix interactions are enhanced, resulting in a better reflection of the physiological microenvironment^[^ ^12^ ^]^; however, they still fall short of recapitulating in a controlled manner the in vivo physiology and pathology of human organ systems^[^ ^13^, ^14^ ^]^ as the previous ones are not cultured in dynamic condition. As we go further through drug screening platforms, although animal models enable in‐vivo analysis, two critical factors reduce the precision and reproducibility of their experimental results: the species differences both between animal and human physiological mechanisms, and between the complexity of their in vivo physiology.^[^ ^13^, ^14^, ^15^ ^]^

Due to the pharmaceutical industry's continuous search for a reliable and efficient framework for drug discovery, recent advances in microfluidics‐based techniques have been made. This resulted in the development of microphysiological systems mimicking the biology and functionality of human organs, also known as organ‐on‐a‐chip (OOC). OOC approaches will improve the modeling of organs and organ systems within healthcare research applications and precision medicine.^[^ ^16^ ^]^ By utilizing OOC technology, scientists can engineer the necessary cytokines, growth factors, nutrients, extracellular matrix (ECM) components, and junction proteins to model the desired tissue.^[^ ^17^, ^18^, ^19^ ^]^ There are two main aspects related to OOC fabrication: the biological model and the chip setup. Mostly, 2D cell models were used in primary OOCs and their behavior was investigated under dynamic condition in comparison to the previously used static culture systems. Although the need for simulating fluid flow circumstances and applying shear stress on the cells is resolved in this aspect, more complex and biologically relevant models in OOCs are demanded that could be achieved by using advanced biofabrication techniques. As chip fabrication point of view, commonly OOCs are fabricated with silicone‐based organic polymers such as polydimethylsiloxane (PDMS) using the soft lithography technique. The fabricated chip would have a compact size with its microchannels precisely patterning cells; furthermore, for controlling cell culture conditions, it would possess the ability to manipulate various fluidic and chemical parameters, such as flow rate, pressure, oxygen, and pH.^[^ ^15^ ^]^ This technique has high‐resolution and accurate control of small features; however, it requires extensive human work and high technical skills, making it costly and challenging to handle.^[^ ^12^ ^]^ As a result, researchers are investigating advanced fabrication methods to simplify this process and enable high throughput.

In the past decade, a dramatic expansion of research into OOCs has converged multiple previously disparate technologies to bring the field closer to its final goal of being commissioned as a drug screening platform that can replace animal studies by addressing aforementioned challenges. Among these methods, 3D bioprinting has gained attention due to its processing versatility, as facilitating the fabrication of desired pore design and interconnectivity is too demanding for conventional scaffold creation methods.^[^ ^20^, ^21^ ^]^ Recently, combining bioprinting and microfluidics has been used to engineer advanced OOCs. Functional artificial tissues and organs would be created by complementing these approaches with these chips achieving preferred complex architectures via layer‐by‐layer assembly.^[^ ^22^ ^]^ Due to the profound influence of the human body's high integrity and dynamic environment on tissue functions, engineered tissue constructs incorporated into the microfluidic system are required to simulate these inter‐organ responses and interactions in OOCs.^[^ ^23^, ^24^ ^]^ The integration of 3D‐bioprinting with microfluidics will allow researchers to build diverse complex structures and adopt multi‐materials. This then enables the fabrication of OOCs into more complex structures with a controllable system that mimics the natural functions of human tissues or organs.^[^ ^25^, ^26^ ^]^

In this review, we investigate the state‐of‐the‐art 3D bioprinting integrated with OOC to biofabricate biomimetic devices. First, different in‐vitro traditional drug testing platforms are introduced with their strengths and weaknesses. Then the bioprinting approach is classified into three main subgroups: direct cell printing, constructing 3D structures and simulating biological barriers, resulting in advanced OOC fabrication. Also, utilizing additive biofabrication techniques for chip fabrication and its advantages are deliberated. Regarding these aspects, the most recent significant advances, such as fabricating 3D complex and vascularized structure on chips, as well as potential and future perspectives of this integrated technology are discussed.

## 2 In Vitro Drug Screening and Disease Modeling Platforms

**Figure 1:**
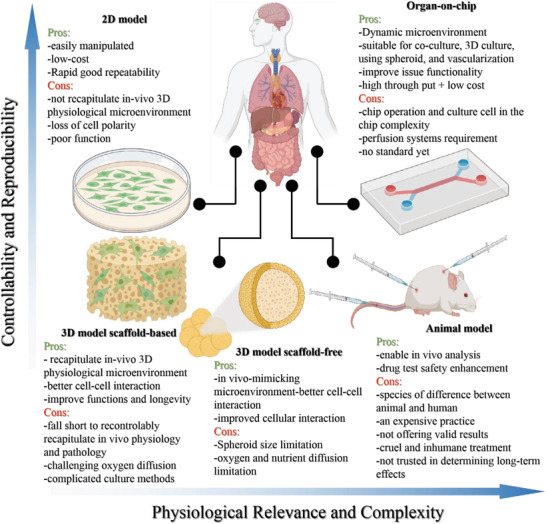
Comparison of different in vitro and in vivo models and their advantages and disadvantages (graphic created with BioRender.com).

Various platforms have been used to investigate new drug development and disease mechanisms: 2D cell models, 3D cell models, OOCs, and animal models. Each of these platforms has its pros and cons, playing a crucial role in selecting a particular platform design depending on the context of its use, as is illustrated in **Figure** **1**. As shown, 2D cell models offer high repeatability, but lower complexity compared to other models. As examples for more complex platforms, 3D cell cultures, both scaffold‐based and scaffold‐free, are considered. Utilizing these models would fall short in real tissue relevancy as they are cultured in a static environment that is unlike the fluid dynamics of the human body. Animal studies are currently one of the most complex methods employed to conduct drug screening and disease modeling. However, there are some challenges hindering investigation in this area such as ethical issues, differences between animal species and inherent mismatch between animal and human physiology. Mentioned obstacles raise the need for an in vitro model that could fulfill the need for an effective drug screening platform. OOCs could be a potential in vitro model addressing those requirements, as they are able to utilize human‐derived tissue models in a dynamic environment similar to the human body condition. In what follows we will elaborate on in vitro drug testing models.

### 2.1 Traditional Drug Screening Methods

To date, 2D cell culture and animal models are the most reliable platforms used in clinic for drug toxicity investigations. 2D in vitro screening is a mainstay of early preclinical development. As apparent, 2D cell culture is the most simple and reproducible model for toxicity measurement. Additionally, animal models are more complex than 2D cell cultures, representing more features of human physiology, however they still do not recapitulate all the necessary features of the human body. In what follows, we will elaborate more on these models.

#### 2.1.1 2D Cell Culture

Due to its high speed and ease of handling, possessing high level of productivity and stability, the conventional 2D cell culture approach has been widely used. It is a convenient and relatively inexpensive technique for diverse biological applications such as drug cytotoxicity evaluation.^[^ ^27^, ^28^ ^]^ Despite these qualities, there are several significant drawbacks of 2D models due to differences between cell behavior in 2D and 3D environments. 2D‐cultured cells lose their intrinsic biochemical cues and the cell‐cell interactions necessary to activate certain signaling pathways. As a result, this method fails to maintain the physiological phenotype and cannot fully recapitulate the intended tissue's specific functions.^[^ ^29^ ^]^ 2D cell culture is unable to mimic the 3D organization of in vivo tissues and organs. Namely, they do not reach sufficient expression levels of ECM proteins such as collagen or fibronectin and demonstrate poor ECM‐cell interactions. In addition, gene expression of cells is different from that of in‐vivo tissues. For drug screening investigations, the absence of drug penetration barriers and drug resistance make 2D cell cultures a weak platform to rely on.^[^ ^30^ ^]^ Therefore, utilizing more relevant models which mimic in‐vivo conditions of human tissues is a priority in drug development.

#### 2.1.2 Animal Models

Along with 2D cell culture platforms for preclinical drug development experiments, animal models have been used in pharmaceutical and industrial research to predict human toxicity. Although utilizing animal models has significant benefits in research and undoubtedly in human being's life quality, however analysis suggests that animal models poorly predict drug safety in humans. Animal research is highly expensive, and may delay the drug approval process. Human subjects have been harmed in the clinical testing of drugs that were deemed safe by animal studies.^[^ ^3^ ^]^ Overall, approximately 89% of newly developed drugs fail human clinical trials, and among them around 50% of those failures are due to unanticipated toxicity in human trials. This statistic thus indicates that animal tests can not accurately predict human toxicity.^[^ ^3^ ^]^ Increasingly, investigators are questioning the scientific merit of animal research and thus searching for more relevant methods for drug screening.

### 2.2 Emerging Drug Screening Methods

Due to the aforementioned drawbacks and limitations of traditional methods, is it time to rethink our current approaches? All in all, it seems that there is an urgent need to develop more biologically relevant platforms as described in the following.

#### 2.2.1 3D Cell Culture

One of the primary steps from 2D cell culture through more relevant models could be culturing cells in a 3D environment. Although 2D cell cultures are still widely utilized, 3D cell culture strategies are starting to catch up. One of the first 3D cultures was made in a soft agar solution by Hamburg and Salmon in the 1970s.^[^ ^31^ ^]^ Culturing cells in a 3D environment would create more relevant cell models, including cell–cell and/or cell–matrix interactions. Additionally, establishing barrier tissues, better simulation of in vivo conditions, and a more realistic environment to grow and treat different cell lines are some advantages of choosing 3D cell cultures over 2D methods.^[^ ^10^, ^32^ ^]^ Thus in many respects, there is a strong preference for 3D cell culture over 2D. Cells in 3D structure preserve natural shape and growth rate and remain well differentiated. Additionally, higher rates of resistance for drug‐induced apoptosis and accurate representation of response to environmental stimuli can be achieved in 3D cell cultures.^[^ ^33^ ^]^ 3D cell cultures can be roughly classified into scaffold‐free and scaffold‐based techniques.^[^ ^34^ ^]^

##### Scaffold‐Free 3D Cell Culture

Scaffold‐free 3D cell culture allows the embryogenesis of cells to grow and create different tissues without the presence of any scaffolds. During embryonic development, cells undergo biological self‐assembly to form complex 3D structured tissues and intensive cell–cell contacts in order to maintain intracellular functions.^[^ ^35^ ^]^ These cells are within an ECM that provides defined biomechanical and biochemical cues needed for determining cell differentiation, proliferation, and homeostasis.^[^ ^34^, ^36^ ^]^ Numerous studies have investigated single‐cell suspensions that will spontaneously self‐assemble to form spherical microtissues in the absence of an ECM or any scaffolds^[^ ^37^ ^]^; these spheroids have been utilized for drug development in several studies.^[^ ^38^, ^39^ ^]^

##### Scaffold‐Based 3D Cell Culture (3D‐Bioprinting)

In contrast with the scaffold‐free 3D cell culture, scaffold‐based systems would result in better manipulation of cell cultures, in terms of position, geometry, and size. However, they commonly cause concerns about biocompatibility and clinical application as generally non‐human, exterior materials are employed to generate scaffolds.^[^ ^40^ ^]^ By selecting a 3D cell culture scaffold for biomedical applications, material properties must be considered because they define physical factors such as culture porosity, stiffness, and stability, as well as biological factors such as cell compatibility or adhesiveness.^[^ ^41^ ^]^ There are several methods of fabricating 3D scaffolds using biomaterials with the desired characteristics and features needed for a particular application. These scaffold fabrication methods can be subdivided into conventional or rapid prototyping methods. Freeze‐drying, gas forming, electrospinning, and thermal‐induced phase separation are examples of conventional techniques, whereas different types of 3D printing are examples of rapid prototyping.^[^ ^42^ ^]^ Through these methods, previous studies have investigated 3D cell culture systems for drug discovery applications.^[^ ^43^, ^44^, ^45^ ^]^

3D cell culture models are expected to yield higher predictive and reliable results for clinical outcomes, but they are still limited in their application for high‐throughput screening and high‐content screening.^[^ ^46^, ^47^ ^]^ Furthermore, the fact that they are cultured in static media necessitates more advanced emerging techniques such as OOCs. Nevertheless, these challenges associated with 3D cell culture systems can potentially be overcome with the assistance of microfluidic technology.

#### 2.2.2 Organ‐on‐Chips (OOCs)

As the result of development in microfabrication and microfluidic devices, OOC models emerged for the first time in 1990 as miniaturized total chemical analysis systems.^[^ ^17^, ^23^, ^48^ ^]^ They serve as powerful new instruments for filling the translational gap between animal models and human disease. They especially have the potential to replace animal trials in the future by reflecting the structural, microenvironmental, physiological and biological functions of human organs on microfluidic chips.^[^ ^16^, ^49^, ^50^ ^]^

Since the early 2000s, researchers have endeavored to develop various microfluidic devices and integrate them with biological building blocks, such as cells or spheroids, to enable controllable and organotypic cell culture for in vitro biochemical and pharmacological analyses.^[^ ^23^, ^51^ ^]^ In 2010, the Ingber group attracted tremendous attention from both biology and engineering communities by reporting a lung‐on‐a‐chip model.^[^ ^52^, ^53^ ^]^ Since then, numerous single‐organ chips have been developed to investigate disease mechanisms and analyze adverse drug reactions, which will be further discussed in the following sections. In addition to single‐OOCs focusing on mimicking individual organ functions, they have also gained attention for generating more reliable data by integrating multiple organ units. This is relevant when replicating in vivo conditions because there is significant crosstalk between different human organs and systems, such as between the gut, liver, and kidney for drug absorbance, metabolism, and elimination, respectively.^[^ ^54^, ^55^ ^]^ By employing OOCs, a high level of control will be achieved, enabling a customized cell‐culture environment. This includes custom ECM topology, the integration of sensors and actuators for monitoring and electrical/mechanical stimuli, control of microfluidic channel dimensions, and temporal and spatial flow profiles for pulsatile flow and chemical stimuli.

In conclusion, through various drug testing platforms, OOC is showing promising potential to become a dominant drug screening device in the future. There are several biofabrication methods which can be used to construct OOCs. To approach high throughput, rapid, reproducible, and scalable methods, 3D bioprinting would be an ideal technique to be incorporated with OOC fabrication steps.

## 3 Integration of 3D‐Bioprinting and OOCs

To successfully replace the use of animal studies and human trials with OOCs for drug evaluation, there is a need for advanced biofabrication techniques which can accommodate most biomimetic tissues or organ models within the chip. Bioprinting is a game‐changing technology that relieves the deposition of biomaterials, biochemicals, and cells in an engineered structure via an automated dispensing system to assemble tissue models.^[^ ^20^, ^56^, ^57^ ^]^ 3D bioprinting enables the creation of several complex structures through a layer‐by‐layer deposition process that adopts various biomaterials and cell lines tailored to the goal tissue.^[^ ^58^ ^]^ This technology is an outstanding method in tissue engineering for creating 3D scaffolds with patient‐specific shapes and complicated designs to create living tissue constructs.^[^ ^59^ ^]^

**Figure 2:**
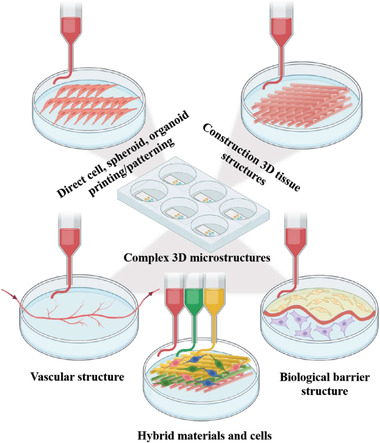
Different approaches for bioprinting within OOCs' chip (graphic created with BioRender.com).

With the convergence of 3D bioprinting and OOC fabrication process, we can create more complex artificial tissues with the proper microarchitecture for mechanical and chemical stimuli. Furthermore, by utilizing hybrid cell lines and biomaterials, an advanced platform that performs human‐like tissue functions could be created. In doing so, 3D printing technology has the capacity to lead OOC engineering into the next generation.^[^ ^58^ ^]^ There are several main approaches used to integrate the 3D bioprinting and OOC fabrication process, as illustrated in **Figure** **2**. In what follows, we will review some specific aspects of 3D bioprinting methods.

### 3.1 Direct Cell, Spheroid, Organoid Printing/Patterning

**Figure 3:**
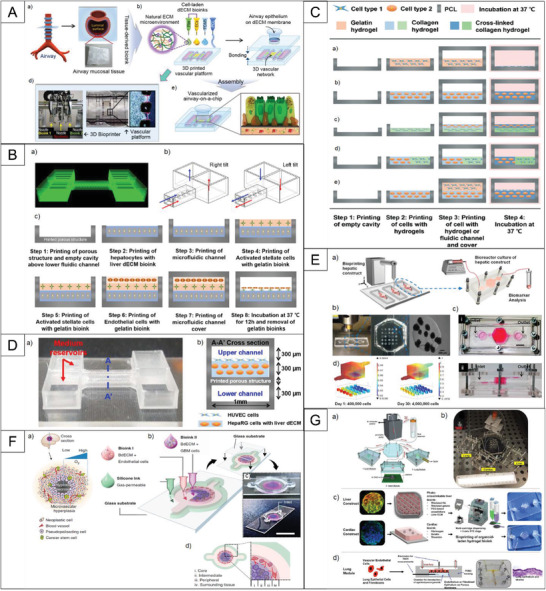
Examples which showcase the use of direct cell, spheroid, and organoid printing/patterning methods. A) Fabrication of vascularized airway‐on‐a‐chip (VA‐OC) by 3D cell printing showing the printing process and the final microstructure of airway and mucosa tissue. Reproduced with permission.^[^ ^63^ ^]^ Copyright 2018, IOP Publishing. B) 3D Liver Fibrosis‐on‐a‐Chip Development using layered structure, a) Printing code for the chip platform. b) Media supply through two different medium reservoirs and microfluidic channels using gravity‐induced force. c) Overall cell‐printing process for the three different cell types for 3D liver fibrosis‐on‐a‐chip printing. Reproduced with permission.^[^ ^64^ ^]^ Copyright 2020, The American Chemical Society. C) 3D bioprinting process (vertical section, side view) of various organ‐on‐a‐chip platforms Reproduced with permission.^[^ ^65^ ^]^ Copyright 2016, The Royal Society of Chemistry. D) Cell‐printed 3D liver‐on‐a‐chip mimicking liver microenvironment, a) Cell‐printed 3D liver‐on‐a‐chip with dual fluidic condition, b) cross‐sectional view of A‐A′ possessing upper and lower channels. Reproduced with permission.^[^ ^66^ ^]^ Copyright 2019, IOP Publishing. E) liver‐on‐a‐chip platform with bioprinted hepatic spheroids using photocrosslinkable GelMA hydrogel‐based hepatic construct within the bioreactor as a dot array.^[^ ^67^ ^]^ Copyright 2016, IOP Publishing F) Working principles of the GBM‐on‐a‐chip confirmation of reconstituted GBM ecology utilizing various bioinks and other materials to construct a compartmentalized structure. Reproduced with permission.^[^ ^69^ ^]^ Copyright 2019, Springer Nature. G) Overall design and implementation strategy for the 3‐tissue‐representative organ‐on‐a‐chip system using a variety of biofabrication approaches. Reproduced with permission.^[^ ^72^ ^]^ Copyright 2017, Springer Nature.

Traditionally OOCs were cell‐seeded inside the chip's bioreactor either manually or by using syringe pumps.^[^ ^60^, ^61^, ^62^ ^]^ However, this method is slow and relies on a tedious and complex setup of pumps, tubing, and fluidic connections; it also has low throughput and reproducibility. Therefore, direct multiple cell printing or patterning via a bioprinter results in precision and reproducibility enhancement, while assuring high throughput. As one of the most straightforward approaches for integrating 3D bioprinting and OOCs, cell printing and patterning has been used in various studies. Park et al.^[^ ^63^ ^]^ employed tracheal mucosa decellularized extracellular matrix to encapsulate and print endothelial cells and fibroblasts within a designated polycaprolactone frame. This design then gradually drives endothelial re‐orientation and provides a niche that emulates conditions in vivo (**Figure** **3**A). Fabrication of the airway‐on‐a‐chip by 3D cell printing allowed them to control the parameters completely; therefore, the chips with the same effect can be produced efficiently. 3D‐cell‐printed vascularized airway‐on‐a‐chip models made in this work successfully recapitulated relevant pathophysiological responses to the stimulation in vitro.

As another example, Lee et al.^[^ ^64^ ^]^ developed a 3D liver fibrosis‐on‐a‐chip co‐culturing three liver cell types using the cell‐printing technique with gelatin bioinks: hepatocytes, activated stellate cells, and endothelial cells. In this study, each nonparenchymal liver cell type was printed as a multilayer construct, shown in Figure 3B, and their position was like the native liver microarchitecture. As a promising result, the 3D liver fibrosis‐on‐a‐chip exhibited collagen accumulation, cell apoptosis, and reduced liver functions as characteristics of liver fibrosis. In similar research done by Lee et al.,^[^ ^65^ ^]^ a novel fabrication method was investigated that eliminated the need for a secondary cell‐seeding process and resulted in severe protein absorption due to the material used. It also helped overcome difficulties in providing various cell types for spatial heterogeneity in the organs‐on‐chips. Heterotypic cell types and biomaterials were used and positioned at the desired position in order to fabricate various liver‐on‐a‐chips, promoting anticipated mimicry of the natural conditions of the liver as illustrated in Figure 3C. Liver function was significantly enhanced on the 3D‐bioprinted liver‐on‐a‐chip. Additionally, Lee's group added bile flow to simulate the biliary system in their liver‐on‐chip platform.^[^ ^66^ ^]^ A chip including a biliary fluidic channel, when compared to one without a biliary system, induced superior liver‐specific gene expression and functions. Figure 3D demonstrates the structure of the chip made in this work.

Moreover, instead of utilizing individual cells in the bioprinting process, spheroids could be printed in OOCs. Bhise et al.^[^ ^67^ ^]^ developed a liver‐on‐a‐chip platform for the long‐term culture of 3D human HepG2/C3A spheroids for drug toxicity assessment. Employing the 3D bioprinting technique, hepatic constructs of spheroids encapsulated within photocrosslinkable gelatin methacryloyl (GelMA) hydrogel were fabricated. They were then assessed by monitoring the secretion rates of albumin, alpha‐1 antitrypsin, transferrin, and ceruloplasmin during the 30 d culture period (Figure 3E). Additionally, the researchers confirmed the possibility of developing OOCs for the application of drug toxicity analysis, as the response of the liver‐on‐a‐chip platform to acute acetaminophen (APAP) treatment was similar to results seen in animal and in vitro models.

In the case of cancer‐on‐chip (COC), several works have been done. For instance, a 3D tumor array chip was established using electrohydrodynamic 3D‐bioprinting to create droplet arrays of GelMA containing breast tumor cells before being applied for screening of epirubicin and paclitaxel.^[^ ^68^ ^]^ In another study, Yi et al.^[^ ^69^ ^]^ developed a bioprinted glioblastoma‐on‐a‐chip in cancer–stroma concentric‐ring structure containing patient‐derived tumor cells, vascular endothelial cells, and decellularized extracellular matrix from brain tissue (Figure 3F). The resulting structure was used to determine drug combinations associated with superior anticancer drugs. Employing controllable cell printing techniques, Mi and his group developed high throughput printing and a precise cell‐printing method to fabricate COC with breast cancer cells. This method was used to study the ability of cancer cells to migrate, as well as cell responses to different concentrations of paclitaxel.^[^ ^70^ ^]^ In a similar study, microfluidic‐based biosensors were fabricated by combining a digital micro‐mirror device (DMD) and cell printing to investigate the metabolism of EFC (7‐ethoxy‐4‐trifluoromethyl coumarin) within the MDA‐MB‐231 (human breast adenocarcinoma) cell line.^[^ ^71^ ^]^ A cell‐laden microfluidic chip was fabricated in the same group by co‐culturing HepG2 and MDA‐MB‐231 cell lines based on extrusion bioprinting. The fabricated COC was used to evaluate cell viability and metabolic activity. Eventually, the final goal of OOC technology research is to integrate them into a human‐on‐chip (HOC). In this direction, Skardal et al.^[^ ^72^ ^]^ fabricated liver‐OC, heart‐OC, and lung‐OC using their organoids embedded in related ECM, then combined them to investigate inter‐organ responses to drug administration (Figure 3G).

In conclusion, cell printing can be divided into two categories of strategies: using bioinks and solely printing cell suspension. In the case of bioinks, the type of biomaterial used will determine the printing technique. When utilizing photosensitive biomaterials, such as GelMA and polyethylene glycol diacrylate, SLA would be the best option. Physical or chemically curable biomaterials may also be used to pattern the cells with an extrusion bioprinter. The latter strategy would have to be carried out mostly by extrusion printing. In this case, cell suspension can be used to print different patterns or sacrificial biomaterials, such as gelatin,^[^ ^65^ ^]^ to allow for shear‐thinning behavior. The result is a reduction in cell death during the printing process. Following incubation and gelatin extraction, these cells will remain in the originally printed pattern.

### 3.2 Construction of 3D Tissue Structures

**Figure 4:**
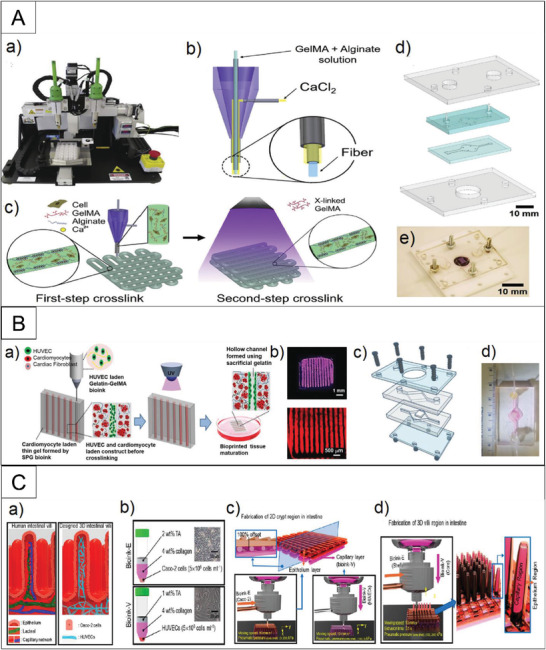
An illustration of key strategies employed to fabricate 3D tissue models. A) Photograph of an Organovo Novogen MMX bioprinter, printed structure and the chip module Reproduced with permission.^[^ ^73^ ^]^ Copyright 2016, Elsevier. B) Embedded 3D bioprinting method for creating vascularized cardiac tissue constructs via embedded bioprinting method Reproduced with permission.^[^ ^74^ ^]^ Copyright 2020, Wiley‐VCH GmbH. C) Intestinal villi model with blood capillaries, a) Designed 3D intestinal model. b) Schematics of bioinks and optical images of Caco‐2 cells and HUVECs. Schematics of fabrication procedures of the c) 2D crypt region and d) 3D villus region for cell‐laden intestinal villi Reproduced with permission.^[^ ^75^ ^]^ Copyright 2018, The American Chemical Society.

In addition to printing and patterning cells, as was elaborated in the previous section, 3D structures can be fabricated utilizing 3D bioprinting. This technique allows deposition of cell‐laden biomaterial, cell spheroid, or tissue strands to form complicated 3D structures within the chip's bioreactor.^[^ ^57^ ^]^ Zhang et al.^[^ ^73^ ^]^ encapsulated endothelial cells within the bioprinted microfibrous lattices to induce their migration towards the peripheral direction of the microfibers and form a confluent endothelium layer. They then seeded with cardiomyocytes, starting the myocardium formation with improved alignment, as shown in **Figure** **4**A. They employed the fabricated structure in endothelialized‐myocardium‐on‐a‐chip for cardiovascular drug testing and endothelialized‐human‐myocardium‐on‐a‐chip as a step toward personalized medicine. Mehrotra et al.^[^ ^74^ ^]^ developed a mechanically robust biomaterial‐ink based on non‐mulberry silk fibroin protein. This acted as a supporting hydrogel for encapsulating human induced pluripotent stem cell‐derived cardiac spheroids (hiPSC‐CSs) and created perfusable vascularized channels via an embedded bioprinting technique. The constructed tissue was used to generate an endothelialized myocardial tissue‐on‐a‐chip as a potential drug screening platform for personalized medicine. Figure 4B illustrates the printing schematics and chip structure.

In another study, Kim and Kim^[^ ^75^ ^]^ employed the 3D bioprinting technique to develop an intestinal villi model, including an epithelium layer and a blood capillary structure. Using two collagen‐based bioinks laden with Caco‐2 cells and human umbilical vein endothelial cells (HUVECs), the epithelium and capillary network of the 3D model were fabricated, as presented in Figure 4C. They stated that the fabricated 3D intestinal villus model with the capillary network would be a platform with high potential for tissue engineering and gut‐on‐a‐chip applications. Additionally, Bowser et al.^[^ ^76^ ^]^ implemented digital projection lithography set up for fabrication of 3D hydrogel‐based neural culture constructs and spheroids made of dissociated rat spinal cord tissue. These were aggregated using magnetic nanoparticles, then bioprinted into the hydrogel constructs using their magnetic properties to control placement. Their results demonstrated successful integration of 3D bioprinting in the microchip industry.

### 3.3 Creating Complex 3D Microstructures

The complexity of all tissues and organs within the human body, coupled with confounding factors associated with injury or disease mechanisms underlying the need for repair, is still a challenge that traditional engineering approaches face. As not all tissues are created equal, these construct designs should first take into consideration concerns involving the various biomaterials and co‐cultured different cell lines required, as well as other biological factors.^[^ ^77^, ^78^ ^]^ Therefore, bioprinting is a promising method being used tremendously to address most of the aforementioned issues.

#### 3.3.1 Employing Hybrid Materials and Cells

3D‐bioprinting as a biofabrication method has great potential for synthetic scaffolds in regenerative medicine. The advantages of fabricating scaffolds using 3D bioprinting are numerous, namely the ability to mimic the anatomical structures of organs and tissues with complex geometries and porosities, using hybrid materials and cell lines most similar to those in actual tissues.^[^ ^79^, ^80^ ^]^ As a significant challenge with OOC techniques, mimicking human tissues and organs is one of the priorities of recently published research using integration with 3D bioprinting.

#### 3.3.2 Vascular and Biological Barrier Structures

**Figure 5:**
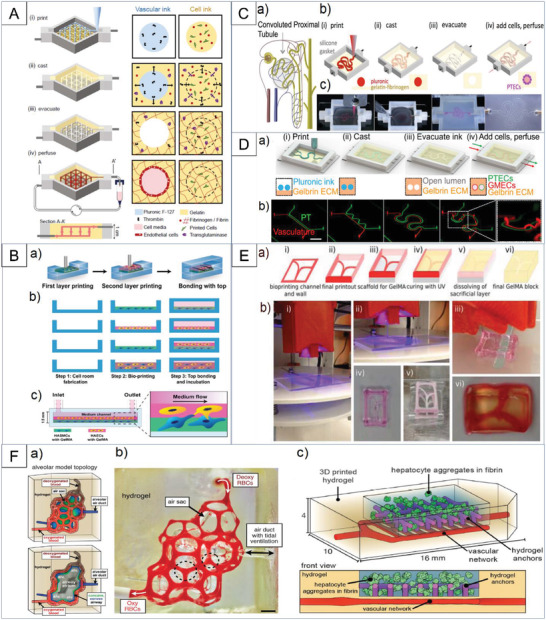
Summary of 3D bioprinting methods which address vascularization challenges associated with the use of tissue models. A) 3D vascularized tissue fabrication using fugitive ink containing pluronic and thrombin, and cell‐laden ink as the matrix for the perfusable channels Reproduced with permission.^[^ ^84^ ^]^ Copyright 2016, National Academy of Sciences. B) Bioprinting two layered cells on microfluidic chip illustrating layered structure vessel‐on‐a‐chip model using cell‐laden hydrogels Reproduced with permission.^[^ ^85^ ^]^ Copyright 2020, Springer. C) 3D convoluted renal proximal tubule on chip corresponding schematics and images of different steps in the fabrication of 3D convoluted, perfusable proximal tubules Reproduced with permission.^[^ ^86^ ^]^ Copyright 2016, Springer Nature. D) Design and fabrication of 3D vascularized proximal tubule models (Scale bar: 10 mm.) Reproduced with permission.^[^ ^87^ ^]^ Copyright 2019, National Academy of Sciences. E) Sacrificial bioprinting of vascularized hydrogels using printed Pluronic template to fabricate hollow channels in GelMA matrix. Reproduced with permission.^[^ ^88^ ^]^ Copyright 2016, The Royal Society of Chemistry. F) Tidal ventilation and oxygenation in hydrogels with vascularized alveolar model topologies Reproduced with permission.^[^ ^92^ ^]^ Copyright 2019, American Association for the Advancement of Science.

Organs in the human body are highly vascularized to allow gas (O_2_, CO_2_) exchange, salts, water, and nutrient diffusion through the tissue and waste disposal^[^ ^81^ ^]^; as a result, these serve as vital biological barriers that separate different tissue compartments.^[^ ^17^ ^]^ Capillary distance within tissues is 60–300 µm, but above this diffusional distance limit, hypoxic regions emerge wherein the survival rates of cells populating in that area dramatically decline.^[^ ^82^ ^]^ With this caveat, a combination of vasculature structures in bioprinted scaffolds is critical for the continuous growth and survival of the enclosed cells. This then allows for the preservation of normal physiological viability and imitation of real organs and tissues.^[^ ^83^ ^]^ With the assistance of bioprinting in advanced microfluidic devices, multiscale hydrogel‐based flow networks can be fabricated with forms and functions close to the real vessels or biological barriers within the body.^[^ ^22^ ^]^ Kolesky et al.^[^ ^84^ ^]^ reported the fabrication of vascularized tissues which exceed 1 cm in thickness and can be perfused on‐chip for long periods (>6 weeks), integrating parenchyma, stroma, and endothelium into a single thick tissue. They coprinted multiple inks containing human mesenchymal stem cells (hMSCs) and human neonatal dermal fibroblasts (hNDFs) within a customized extracellular matrix alongside embedded vasculature. This matrix was subsequently lined with human umbilical vein endothelial cells (HUVECs), as illustrated in **Figure** **5**A. These thick vascularized tissues were perfused with growth factors to differentiate hMSCs toward an osteogenic lineage in situ. In another experiment, Abudupataer et al.^[^ ^85^ ^]^ employed a gelatin‐methacryloyl‐based hydrogel to print a 3D construct for co‐culturing endothelial cells (ECs) and smooth muscle cells (SMCs) on a microfluidic chip. Fabricated EC‐SMC co‐culture chip models upregulated *α*SMA and SM22 protein expression of the SMC to a greater degree and maintained the contractile phenotype of the SMC; this mimicked the niche microenvironment of natural vessels in comparison with the traditional culture system (Figure 5B).

Homan et al.^[^ ^86^ ^]^ developed a kidney model‐on‐chip using a bioprinting method for creating 3D human renal proximal tubules in vitro. It was fully embedded within an extracellular matrix and housed in perfusable tissue chips (Figure 5C) and maintained for more than two months. Their convoluted tubular architecture is circumscribed by proximal tubule epithelial cells and actively perfused through the open lumen. Significant enhancements in epithelial morphology and functional properties were reported in the developed proximal tubules on‐chip compared to the same cells grown on 2D controls, with or without perfusion. Additionally, they introduced the nephrotoxin cyclosporine A to the proximal tubules on‐chip, which resulted in the disruption of the epithelial barrier in a dose‐dependent manner. This bioprinting method provided a promising route for the programmable fabrication of advanced human kidney tissue models on demand. Similar research done by Lin et al. created 3D vascularized proximal tubule models containing two adjacent channels lined with confluent epithelium and endothelium. These were performed as 3D kidney tissue models,^[^ ^87^ ^]^ the fabrication process for which is shown in Figure 5D.

As another example, Zhang et al.^[^ ^88^ ^]^ utilized 3D bioprinting in order to construct a highly biomimetic thrombosis‐on‐a‐chip model, which consisted of microchannels coated with a layer of confluent human endothelium. The endothelium was embedded in the GelMA hydrogel (Figure 5E), where whole human blood was infused and induced to form thrombi. Using continuous perfusion with tissue plasmin activator, non‐fibrotic clots dissolved, revealing the clinical relevance of the model. Further encapsulation of fibroblasts in the GelMA matrix demonstrated potential migration of these cells into the clot and facilitated fibrosis remodeling by subsequent deposition of collagen type I over time. Their study showed the potential of in vitro 3D‐bioprinted blood coagulation models to study the pathology of fibrosis, particularly in thrombosis.

Schöneberg and his group's related research led to the development of perfusable complex vessel chips using drop‐on‐demand bioprinting techniques for generating in vitro blood vessel models.^[^ ^89^ ^]^ The custom‐built bioprinter has three printheads containing HUVECs, SMCs, and thrombin, respectively, for crosslinking to create a vessel about 1 mm in diameter with a wall thickness of about 0.43 mm. Biomimicry of these controllable fluidic systems was greatly improved due to increased expression of essential cues for intercellular communication and tissue formation, such as cadherin and cluster of differentiation 31 (CD31) molecules from the inner layer of HUVECs. Another group created a functional in vitro vascular channel with a perfused open lumen using 3D bioprinting technology, which can support tissue viability up to 5 mm in the distance at 5 million cells mL^−1^ density under the physiological flow condition.^[^ ^90^ ^]^ Bertassoni et al.^[^ ^91^ ^]^ reported a micromolding technique with 3D‐bioprinted agarose as sacrificial template fibers to fabricate various architectural microchannels within GelMA‐based photocrosslinkable hydrogel constructs for applications relevant to regenerative medicine and OOC technology. Additionally, in order to fabricate complex 3D transport regimes that are biophysically and biochemically entangled, Grigoryan et al.^[^ ^92^ ^]^ established intravascular and multivascular designs for both lung and liver models, as demonstrated in Figure 5F, declaring that they have a promising application for lab‐on‐chips and OOCs.

In conclusion, there are two main methods to fabricate hollow channels or barriers within a matrix. The first is utilizing extrusion bioprinting with fugitive inks and the second is directly bioprinting the whole structure with hollow sections inside. Extrusion‐based bioprinting implements pneumatic, piston, or solenoid‐driven devices to force mostly high‐viscosity bioinks through a nozzle and onto a printing surface or in suspending support. The use of extrusion bioprinting methods is common in creating vascular constructs through bioprinting. Generally, this technique has a lower resolution compared to other bioprinting techniques with a minimum feature size around 100 µm.^[^ ^93^ ^]^ Due to limitations in resolution, the printing of capillary networks within structures primarily depends on the natural formation of blood vessels within the filaments following printing. In contrast, large, vessel‐like channels can often be directly or indirectly printed.^[^ ^91^, ^94^, ^95^, ^96^, ^97^ ^]^ Researchers have frequently employed this technique to quickly create larger channels, which are then lined with endothelial cells to produce functional vasculature. Coaxial nozzles and embedded bioprinting are typically used in this technique. Regarding biomaterials being utilized, a variety range of viscosities are suitable. For on‐bed extrusion bioprinting, highly viscose and free‐form standing biomaterials should be used; however, for embedded bioprinting applications, due to the presence of a bath support, less viscose biomaterials and soft hydrogels are more appropriate.^[^ ^98^ ^]^ Stereolithography is another commonly used bioprinting technique for tissue engineering and vascular bioprinting. Biomaterials most well‐suited to this technique are required to be photocrosslinkable. The biomaterial needs to exhibit liquid‐like behavior while in the printing reservoir but then must quickly solidify upon exposure to light. Nguyen and West^[^ ^99^ ^]^ provides a detailed discussion on various photocrosslinkable hydrogels and photoinitiators which are appropriate for stereolithography. Photoreactive acrylate/methacrylate groups can be added to synthetic polymers such as polyethylene glycol (PEG) and polyvinyl alcohol (PVA), as well as natural polymers like gelatin and hyaluronic acid, to enable printing using stereolithography.^[^ ^100^ ^]^

**Table** **1** summarizes the experimental details of studies which used 3D bioprinting for the biofabrication of OOCs.

**Table 1:** 3D bioprinting techniques used to fabricate OOC platforms

Fabrication method	Targeted organ	Printing technique	Chip material	Biomaterials and cells	Major Advance	Chip maintenance	Refs.
Direct cell printing/patterning	Lung	Extrusion	‐PCL ‐PDMS	‐Tracheal mucosa‐derived dECM ‐Human dermal microvascular endothelial cells (hDMECs) ‐Human lung fibroblasts (LFs)	Provides a chemical microenvironment Mimicking the human airway mucosa, and enables modeling of respiratory diseases in vitro by recapitulating inflammatory responses	3 months	[63]
	Liver Fibrosis	Extrusion	Poly‐ (ethylene/vinyl acetate) (PEVA)	‐Gelatin ‐Liver dECM ‐Human HepaRG ‐human umbilical Vein endothelial cells (HUVECs) ‐human LX2 hepatic stellate	‐Exhibited characteristics of liver fibrosis such as collagen accumulation, cell apoptosis, and reduced liver functions ‐Enhanced cell delivery capability	–	[64]
	Liver	Extrusion	PCL	‐Gelatin ‐Collagen ‐ Human hepatocellular carcinoma (HepG2) ‐ HUVECs	Liver function significant enhancement on the 3D bioprinted liver‐on‐a‐chip	–	[65]
	Liver	Extrusion	‐PEVA ‐ Poly (methyl methacrylate) (PMMA)	‐Gelatin ‐Liver dECM ‐ Human HepaRG ‐ HUVECs	‐by adding biliary flow, superior function Compared to 2D/3D culture conditions ‐Sensitive drug response	–	[66]
	Liver	Extrusion	‐PDMS ‐PMMA	‐GelMA ‐HepG2/C3A	Utility as a platform for drug testing, similar response with 15 × 10^−3^ m acetaminophen as animal study	30 d	[67]
	Tumor	Electrohydrodynamic 3D printing	‐Stainless steel ‐Silicon	‐GelMA ‐MDA‐MB‐231 breast tumor cells	A reliable platform for anticancer drug screening	–	[68]
	Tumor	Extrusion	Glass	‐dECM ‐ U‐87 MG cells ‐ HUVECs	Reproducing clinically observed patient‐specific resistances to treatment with concurrent chemoradiation and temozolomide	1–2 weeks	[69]
	Tumor	Drop‐on‐demand	‐Glass ‐PDMS	‐ MDA‐MB‐231 human breast cancer cells ‐HUVECs	Demonstrates viability of bioprinting to position individual cells within microfluidic devices by depositing deposit sub‐nanoliter quantities of breast cancer cell suspension	7 d	[70]
	Tumor	Photolithography	‐PDMS ‐ SU‐8 2100	‐ MDA‐MB‐231 human breast cancer cells	3D printing technology was used to fabricate every aspect of the device	‐	[71]
	Tumor	Extrusion	‐PDMS ‐ SU‐8	‐ MDA‐MB‐231 human breast cancer cells ‐HepG2	Uses a maskless printing technology to fabricate all aspects of the microfluidic device	–	[101]
	MultiOOC (liver, cardiac and lung)	Extrusion	‐PDMS ‐PMMA ‐PCL	‐Hepatic stellate cells (HSCs) ‐Primary human hepatocytes ‐Kupffer cells ‐iPSC‐derived cardiomyocytes (iPSC CMs) ‐Liver (hyaluronic acid + gelatin) ‐Cardiac (fibrin‐gelatin) ‐Endothelial cells ‐Lung fibroblasts ‐Lung epithelial cells	Integration of multiple functional human organoids and tissue constructs into single platforms	–	[72]
Construction 3D tissue structures	Heart	Extrusion	PMMA	‐GelMA ‐HUVECs ‐ hiPSC‐cardiomyocytes	‐ Assembly of the endothelial cells Within the bioprinted microfibers resembling a blood vessel structure ‐ Generated perfusable, endothelialized cardiac Organoids capable of modeling the native myocardium for drug‐related cardiotoxicity assays	–	[73]
	Heart	Extrusion	PDMS	‐GelMA ‐ Polyethylene glycol dimethacrylate (PEGDMA) ‐ Silk Fibroin ‐ Neonatal rat cardiomyocyte ‐ Cellartis Human iPS Cell Line 12 (ChiPSC12)	‐ Biomaterial ink: facilitated the fabrication of anisotropic cardiac constructs, promoted the functional attributes of the cardiomyocytes in terms of maturation, maintenance of cytoskeletal structure and beating potential ‐ potential platform for screening several drugs	–	[74]
	Gut	Extrusion	‐	‐Collagen ‐HUVEC ‐ Caco‐2 cells	‐ effectively establishment of cell‐to‐cell interactions by demonstration of junction markers ‐providing efficient microcellular environmental conditions for achieving successful differentiation of the Caco‐2 cells and substantial enhancement of the barrier function	30 d	[75]
	Nerve	Extrusion/Magnetic	‐	‐ Polyethylene glycol diacrylate (PEGDA)	‐ Combination of digital projection lithography and magnetic spheroid bioprinting ‐ Provide a 3D culture environment with a macrostructure that guides and supports long‐distance 3D neural Projections	–	[76]
Creating complex 3D microstructures (Vascular and biological barrier)	vasculature	Extrusion	‐	‐Gelatin ‐Fibrinogen ‐Pluronic F‐127 (sacrificial) ‐Human mesenchymal stem cells (hMSCs) ‐Human neonatal dermal fibroblasts (hNDFs) ‐HUVECs	‐Multimaterial 3D bioprinting thick vascularized tissue ‐Long‐term stability	>6 weeks	[84]
	vasculature	Extrusion	PMMA	‐GelMA ‐Primary human aortic endothelial cells (HAECs) ‐Human aortic smooth muscle cell line CRL1999 (HASMC) ‐NIH/3T3 fibroblast cell lines	‐Establish different vascular cell types and biomaterials at the desired position ‐Provide hydrodynamic and mechanical properties creating a more realistic vascular tissue	–	[85]
	Vasculature (Convoluted renal proximal)	Extrusion	Two‐part silicone elastomer	‐Gelatin ‐ Pluronic F127 (sacrificial) ‐ Proximal tubule epithelial cells (PTECs)	Promote the formation of a biomimetic epithelium with improved phenotypic and functional properties relative to the same cells grown on 2D controls	>2 months	[86]
	Vasculature (Thrombosis)	Extrusion	PDMS	‐GelMA ‐ Pluronic F127 (sacrificial) ‐ HUVECs	‐Biomimetic model in recapitulating the fibrosis process in vivo	14 d	[88]
	Vasculature	Drop‐on‐demand bioprinting	–	‐ Fibrinogen ‐ Human umbilical vein endothelial cells (EC) ‐ Normal human dermal fibroblasts ‐ Human umbilical artery smooth muscle cells (HUASMC)	Native composition of cells and structural integrity of vessel tissue composite	3 weeks	[89]
	Vasculature	Extrusion	Polycarbonate	‐Gelatin ‐Collagen ‐ HUVECs	Model for investigating mechanisms vascular remodeling underflow condition	3 weeks	[90]
	Vasculature	Extrusion	GelMA	‐GelMA ‐ Poly(ethylene glycol‐co‐lactide) acrylate (SPELA) ‐ Poly(ethylene glycol) dimethacrylate 1000 (PEGDMA) ‐ Poly(ethylene glycol) diacrylate 4000 (PEGDA) ‐ Agarose ‐ Mouse calvarial pre‐osteoblasts cells (MC3T3) ‐HUVECs	An effective technique for vascularization of hydrogel constructs	14 d	[91]
	Vasculature	Extrusion	‐Silicone gasket ‐Glass slide	‐Gelatin ‐Fibrinogen ‐ Pluronic F127 ‐PEO ‐ PT epithelial cells (PTECs) ‐ Endothelial cells (GMECs)	Exhibiting selective reabsorption and vectorial transport of solutes	–‐	[87]

All the previously mentioned techniques using 3D bioprinting to fabricate biorelevant OOC devices could be utilized in different circumstances according to the data that is needed. Cell biopatterning is the easiest approach in terms of fabrication process. However, unlike 2D cell cultures, OOCs can simulate dynamic human organ and tissue conditions by expressing the appropriate biomarkers and ECM proteins required to represent real cell behavior. As another approach, capsulating cells in 3D scaffolds under dynamic fluid flow is more representative of human physiology than 2D cell bioprinting, but it still falls short when simulating 3D complex microstructures of the targeted tissue. In the body, drug molecules are first absorbed into the bloodstream after administration, then distributed across all systems via the circulatory system. However, in OOCs, administered drugs are directly introduced to the scaffolds present within the 3D structure. To mimic true in vivo drug metabolism, vascular barriers must be developed inside the 3D scaffolds to increase their complexity, as in the body. Although manufacturing these features is no doubt challenging, requiring precise instruments and approaches, the output data will ultimately benefit from greater accuracy and relevance to human systems.

## 4 Integration of 3D Printing to Fabricate Chips

Various researchers have focused on the biological aspects of tissue models within the bioreactor of the chip; however, fabricating the main chip is an equally critical process for the success of these devices. Conventionally, soft lithography, photolithography, and etching techniques were used extensively to fabricate OOC devices, but they imposed severe limitations which impede the pace of development and innovation within microfluidic applications: 1) a large number of repeated processing steps are required; 2) an inability to create continuously curved structures and complex geometries; 3) long lead times and high labor obligation; 4) high production costs; 5) low production rates; 6) low levels of reproducibility, dimensional accuracy and surface quality; and 7) the need for separate cleanroom facilities and adroit users.^[^ ^102^, ^103^, ^104^ ^]^ Hence, a rapid prototyping technique is needed to address the limitations of conventional methods. 3D printing is potentially a promising solution; not only would it be high throughput, but it could also ensure the capacity for industry applications.

**Figure 6:**
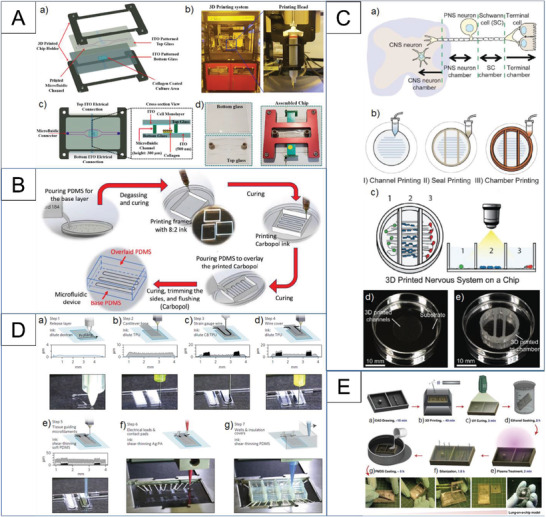
Examples of microfluidic chip fabrication with the use of 3D printing techniques. A) Microfluidic glass chip fabrication illustrating expanded schematic and final chip. Reproduced with permission.^[^ ^105^ ^]^ Copyright 2020, Elsevier. B) Schematic elucidating the concept of using Carbopol gel as a sacrificial ink for fabrication of microfluidic devices in a multi‐step process. Multiple devices can be fabricated on a single Petri dish in a throughput manner. Reproduced with permission.^[^ ^106^ ^]^ Copyright 2019, IOP Publishing. C) Schematic of the nervous system, modeled as four primary components: CNS neurons, PNS neurons, axon‐associated Schwann cells, and epithelial cells, and how it is modeled on a chip. Reproduced with permission.^[^ ^107^ ^]^ Copyright 2016, The Royal Society of Chemistry. D) Device principle and microscale 3D‐printing procedure of the heart‐on‐a‐chip. Reproduced with permission.^[^ ^109^ ^]^ Copyright 2016, Springer Nature. E) Protocol for the surface treatment of the 3D printed resin molds. Reproduced with permission.^[^ ^103^ ^]^ Copyright 2019, Elsevier.

Khalid et al.^[^ ^105^ ^]^ developed a multisensor lung cancer‐on‐chip platform for cytotoxicity evaluation of doxorubicin and docetaxel as drug candidates. They used an inkjet 3D‐printing system to print the microfluidic channel on the bottom of the top glass and chip holder to hold the top and bottom glasses together, as demonstrated in **Figure** **6**A. In another study, Ozbolat et al.^[^ ^106^ ^]^ investigated Carbopol as a sacrificial gel to create microfluidic channels in PDMS devices. Figure 6B shows that the printed channel was overlaid with PDMS to create microfluidic devices upon curing PDMS and removing the sacrificial Carbopol ink. Johnson et al.^[^ ^107^ ^]^ introduced a 3D printing approach in the form of a bioinspired, customizable 3D printed nervous system on a chip to investigate viral infection. Micro‐extrusion 3D printing strategies enabled fabricating biomimetic scaffold materials to construct microchannels and compartmented chambers to align axonal networks and spatial arrangement of cellular components (Figure 6C).

Skardal et al.^[^ ^108^ ^]^ also employed a 3D printing approach to fabricate the main chip, which consisted of two circular chambers. The inverted chamber/channel structures were 3D‐printed from molds made of polydimethylsiloxane. In a high‐quality study, Lind et al. utilized 3D printing for almost all chip component manufacturing.^[^ ^109^ ^]^ Through their 3D printing method, six inks were used, each with a different purpose. Some inks, such as dextran, thermoplastic polyurethane, and shear‐thinning PDMS, were used as substrates and self‐assembly cues; another one, the carbon black in thermoplastic polyurethane and silver‐laden polyamide, composed the soft electrical parts of the system. The six inks were used to create three separated components (the base, the sensor, and the tissue‐guiding layer), serving to support, measure, and manipulate cells on the surface. The process of chip fabrication is illustrated in Figure 6D. This microphysiological device facilitated tissue culture, and by using sensors, the non‐invasive analyses of tissue contractile strength could occur over several weeks.

Some studies have employed 3D printing techniques for constructing molds which can fabricate OOC devices. This technique can increase scalability and lead to mass production for industrial means. Lantada et al.^[^ ^110^ ^]^ presented a laser stereolithography process that enables the rapid prototyping of 3D multiscale mold cavities for the industrialized mass production of OOCs. Similarly, Shrestha et al.^[^ ^103^ ^]^ have used digital light processing (DLP) 3D printing technologies with a resolution of 30 µm for the rapid fabrication of molds for PDMS casting (Figure 6E). To prevent the PDMS from sticking to the inside of the cast, the surface was treated by post‐curing the mold after being washed with isopropanol. They utilized the fabricated chip for a lung‐on‐a‐chip model.

**Table** **2** summarizes the studies which used 3D printing for the biofabrication of OOC chips.

**Table 2:** 3D printing techniques used to fabricate OOC platforms

Device	Printing method	Chip fabrication material (s)	Main observations	Refs.
Lung cancer‐OC	Inkjet	‐ Biocompatible silicone elastomer ‐PDMS	Lung cancer‐on‐chip system integrated with biosensors for real‐time monitoring of physiological events	[105]
Vessel‐OC	Extrusion	‐ Carbopol gel ‐PDMS	A highly affordable and practical approach for PDMS devices manufacturing with closed fluid channels	[106]
Nervous system‐OC	Extrusion	‐ Silicone ‐ Polycaprolactone (PCL)	Providing automated assembly of chambers and customized design of the microchannels and the chamber geometry	[107]
Metastasis‐OC	Extrusion	PDMS	Study the translocation of metastatic tumor cells from the primary tissue site to the downstream tissue site	[108]
Heart‐OC	Extrusion	‐ Dextran ink ‐ Thermoplastic polyurethane (TPU) inks ‐ Ag:Pa ink ‐ Soft PDMS Ink ‐ Rigid PDMS ink	‐ Automated design and fabrication of instrumented cardiac microphysiological devices ‐ Leveraging the ability to track the temporal development in tissue mechanics	[109]
Multi‐organ‐OC	Laser stereolithography (SLA)	Epoxy resin	Designing and rapid mass production of OOC devices	[110]
Lung‐OC	Digital light processing (DLP)	Epoxy resin	‐Robust, cost‐effective, and simple technique to fabricate chips in less than a day ‐The molds can also be utilized for repeated PDMS casting	[103]

Previous studies have shown that additive manufacturing would lower the cost, time, and labor requirements needed for conventional microfluidics fabrication methods. Additionally, combining traditional techniques with 3D printing, or using different methods of additive manufacturing, could potentially result in more efficiency when constructing more complex structures.

## 5 Application of OOCs

This section will focus on the OOCs various applications in the biomedical field. One of the potential applications is evaluating drug safety and toxicity prior to clinical trials. Furthermore, “personalized medicine,” a newly emerging technology, describes the optimal matching of drug regimens to individuals and genetically unique patients. Personalized medicine attracts significant attention in drug development research to minimize side effects and costs for new and existing drugs; OOCs could be used to achieve this goal. Prior to drug design and screening, drug metabolism investigation and organ disease modeling are useful to understand diseases deeply and gather fundamental information for drug development process. This lends OOCs a critical role in gathering the required fundamental information important to the drug development process.

### 5.1 Drug Screening and Development

Although they may not show adverse effects on animals during the preclinical stage, several newly developed drugs can unpredictably cause different organ impairments in patients during clinical trials.^[^ ^13^, ^111^ ^]^ Therefore, OOCs derived from human cells can serve as reliable, efficient, and accurate platforms for drug toxicity assessment before the molecule is approved for clinical trials. For instance, lung cancer‐OC described in previous sections was used to evaluate the cytotoxicity of two drug models, doxorubicin and docetaxel.^[^ ^105^ ^]^ Utilizing pH sensor and transepithelial electrical (TEER) impedance, more toxicity was found for doxorubicin in comparison to docetaxel as the concentration increased.

### 5.2 Personalized Medicine

Currently, heterogeneity in the progression of many diseases is widely observed. This suggests the need for treatment, monitoring, or prevention strategies which are designed to be customized or “personalized” to that individual's distinctive biochemical, physiological, environmental, and behavioral profile.^[^ ^112^ ^]^ Today, personalized medicine is a novel and exciting field, attracting attention in the medical and healthcare industries. Personalized medicine would be a promising concept to transform medical interventions by providing practical, tailored therapeutic strategies based on an individual patient's unique genetics, whilst also remaining mindful of their personal situation and environmental factors. The power of personalized medicine lies not only in treatment but in prevention. For instance, increased utilization of molecular classification of patients to screen for mutations that can give rise to resistance against specific treatments provides reliable evidence for medical professionals to tailor treatment strategies. This approach minimizes adverse outcomes which often arise as a result of trial‐and‐error prescribing methods.^[^ ^113^, ^114^, ^115^ ^]^ OOCs as trustworthy time‐ and cost‐saving platforms for drug screening could be used for personalized medicine research and development.^[^ ^116^ ^]^ For example, in the case of glioblastoma‐OC (GBM‐OC), Yi and colleagues printed GBM‐OC with primary GBM cells obtained from patients showing very different manifestations in the clinic, then evaluated any resistance demonstrated by the patient‐derived GBM cells against the treatment.^[^ ^69^ ^]^

### 5.3 Drug Metabolism Investigation

Drug metabolism plays a critical key role in determining drug efficiency and potential side effects.^[^ ^117^ ^]^ There has been extensive research interest in using advanced cell‐based methods to predict how toxic a pharmaceutical or chemical will be, as well as the mechanisms by which it might be metabolized in target organs. Except for various animal models used for these studies,^[^ ^118^, ^119^ ^]^ OOCs have shown reassuring potential for drug metabolism investigations.^[^ ^120^, ^121^, ^122^ ^]^ For example, one such study used a 3D‐printed tumor‐OC to understand the relative pharmacokinetic efficiency and interconnected microfluidics for the compound 7‐ethoxy‐4‐(trifluoromethyl)coumarin (EFC). The results demonstrated that the metabolism process converts EFC to 7‐hydroxy‐4‐(trifluoromethyl)coumarin (HFC) by the enzyme 7‐ethoxycoumarin O‐deethylase.^[^ ^71^ ^]^

### 5.4 Organ Disease Modeling

Disease models using microphysiological systems (MPS) open new routes for understanding pathologies and potential treatments. These diseases could be modeled with primary or induced stem cell sources from patient donors, or by using genetic tools to induce a disease phenotype.^[^ ^123^ ^]^

Additionally, treatment options for patients with a rare disease remain a considerable challenge; fewer than 5% of approximately 7000 rare diseases identified to date have known and effective drug therapies.^[^ ^124^ ^]^ In this case, employing OOC technology may provide a deeper understanding of many currently unknown disorders. Moreover, this method provides new drug screening devices for existing therapeutics and repurposes existing drug testing platforms.^[^ ^125^ ^]^

Regarding their many avenues for application in medicine and biotechnology, OOCs have great potential to be a leading platform for drug screening and personalized medicine in the future. However, at the current stage, OOCs are still limited in their ability to achieve the most relevant conditions similar to that of human tissues and organs. Therefore, further research should concentrate on efficient and effective methods of constructing biomimetic and reliable tissues on chip.

## 6 Challenges and Future Direction

Recently, organ‐on‐a‐chip has received attention with their promising potential to be substituted for animal studies in drug development. The synergistic combination of bioprinting technology and microfluidics has the potential to improve physiological models and their reliability. Though there have been many outstanding results and outcomes reported in the literature using this multidisciplinary technology, several obstacles remain prohibiting their extensive use in drug development. These obstacles can be classified into two main types: bioprinting‐related issues and OOC‐related issues.

The challenge of developing bioink with the most appropriate biomaterial is one example of a bioprinting‐related obstacle. Bioinks should simultaneously have proper physical properties for the 3D micropatterning of living cells capsulated within them and biological cues for the in vitro regeneration of target tissues. Because most of the materials used for printing do not inherently possess all these properties, they would not produce high‐quality bioink. Lately, newly emerged materials known as decellularized extracellular matrix‐based bioinks (dECM‐based) have exhibited good printability and superior tissue‐specific cytocompatibility to encourage cell proliferation and relevant gene expression.^[^ ^126^, ^127^, ^128^ ^]^

Additional to bioinks, bioprinting processes and techniques should be modified to be used in OOCs. Their resolution is one of the crucial factors in constructing biomimetic structures. For instance, as presented in real tissues, bioprinting complex vascular networks is still one of the most critical challenges to producing biomimetic tissues. To date, several research groups have introduced novel processes to create a simplified version of these networks,^[^ ^81^ ^]^ but they were unable to show evidence of their functionality as compared with real organ and tissue systems. To this end, gaining a deeper understanding of cell–cell interactions is essential; further experimentation should be conducted to understand the biological aspects of tissues and mimic their functionality using technology. Besides technical and structural matters, cell sources and co‐culturing them are another difficulty in developing bioprinting‐assisted OOC systems. Furthermore, the difficulty of finding sustainable and reliable sources of cells present a key limitation. Primary cells are the most ideal candidates, although dedifferentiation remains problematic in vitro. A way to overcome this issue is to use mutable human embryonic stem cells and iPSCs as promising alternative sources.^[^ ^129^ ^]^ Additionally, co‐culturing different cell types presents challenges to developing advanced OOC systems. Most studies on bioprinted OOCs focus on a single tissue model, therefore, more research is needed to consider multiorgan models to approach the ultimate goals of OOC systems.

In addition to the bioprinting step, there are several obstacles present in chip fabrication and monitoring. PDMS is one of the most used polymers for main chip body fabrication that faces critical drawbacks. However, if it was used to integrate OOCs with electrodes, there would be difficulty depositing this polymer directly on the surface. Furthermore, another main drawback for cell biology is that PDMS can absorb small hydrophobic molecules, like biomolecules and drugs, from the solution to influence solvent efficacy and toxicity. It is thus necessary to replace suitable alternative biologically inert materials to address these issues.^[^ ^130^ ^]^ As another point, most of the fabricated chips lack real‐time dynamic monitoring, ignoring many important physiological processes. Integrating sensors would be a promising solution for real‐time, in situ, and dynamic maintenance and monitoring of OOCs, making them suitable for the rapid high throughput of industrial applications. Various in situ optical, electrical, chemical, and biological biosensors could be incorporated with OOCs to detect key signals; these biosensors have traditionally been studied through off‐chip techniques such as enzyme‐linked immunosorbent assay (ELISA), polymerase chain reaction (PCR), and single‐cell mRNA sequencing (scRNA‐seq).^[^ ^109^, ^131^ ^]^

Another critical technical challenge arises in terms of the commercialization of these devices. Although some companies have shown interest, there are still obstacles limiting wide commercial applications of these devices. To achieve this, we need to optimize the bioprinting step to be capable of biofabrication in a scalable, high‐speed manner that has high throughput and proper resolution to successfully fabricate heterogeneous biomimetic tissue constructs. Furthermore, the high cost of manufacturing and experimental implementation is another vital limitation. To this end, OOC constructs should be miniaturized to minimize the time and cost of the fabrication process whilst retaining appropriate tissue functions.

One of the most important aspects in drug development is to investigate immune responses to that drug. The immune system plays a critical role in drug design and development, as it can impact the efficacy, safety, and overall success of a drug. A drug's efficacy can be impacted by the immune system, wherein the body's immune response can either enhance or inhibit the drug's ability to target its intended target. Understanding the interplay between a drug and the immune system can help to optimize drug efficacy.^[^ ^132^ ^]^ Much of the previous research on the immune system has been carried out either in 2D cell cultures under laboratory settings or through in vivo animal studies.^[^ ^133^, ^134^ ^]^ Furthermore, most in vivo research was initially performed on small rodents, such as mice, before proceeding to the human testing of any therapeutics or treatments. However, there are significant differences between the immune systems of mice and humans, which can lead to detrimental outcomes in clinical studies.^[^ ^135^, ^136^ ^]^ In vitro research using 2D cell cultures fails to accurately replicate the complex physiological environment found in the body, making it unable to accurately reflect the results of in vivo research.^[^ ^137^ ^]^ As a result, researchers have shifted to using 3D systems since the 1980s.^[^ ^44^ ^]^ Human cell‐based 3D systems provide a more realistic simulation of the human body, bridging the gap between traditional 2D cultures and animal models and human clinical trials, while avoiding the ethical concerns associated with animal testing.^[^ ^138^, ^139^ ^]^

The assessment of the immune system using 3D culture systems such as OOC models has become increasingly popular in recent times because of its significance in major diseases and the advancement of therapy methods which leverage the use of antibodies and immune pathways. Recently, several immune system‐on‐a‐chip models have aimed to emulate different aspects of this system such as lymph nodes^[^ ^140^ ^]^ and bone marrow,^[^ ^141^ ^]^ as well as immune responses in some organs including the liver,^[^ ^142^ ^]^ lung,^[^ ^143^ ^]^ skin,^[^ ^144^ ^]^ and gut.^[^ ^145^ ^]^ However, in order to create a relevant immune system‐on‐a‐chip, the biological model should adequately mimic the natural niche of targeted immune organs. One of the most crucial challenges hindering clinical OOC usage is their relevancy and mimicking the physiological aspects of the immune system to generate valid data. For instance, it is imperative to imitate the lymph node's antipathogenic abilities in drug development using a microfluidic platform. However, to the best of our knowledge, there is no lymph node‐on‐a‐chip developed to date that can replicate the complexity of its architecture, which contains many cell types and continuous immune cell migration. Lymphatic vessels play an essential role in the efficacy of immune responses; however, this makes the microstructure challenging to mimic artificially.

The highly vascularized structures of bone marrow also share this same challenge.^[^ ^146^ ^]^ Accordingly, 3D bioprinting, as the main biofabrication method being discussed in this manuscript, could be one of the potential strategies best suited to approach these complexities fabricating immune systems‐on‐a‐chip. Some studies have investigated bioprinting lymph nodes or bone marrow structures, but they have not been tested under a dynamic culture condition. Jin et al. implemented bioprinting to replicate the function of T‐cells by mimicking a 3D microenvironment within lymphatic vessels and lymph nodes in vitro.^[^ ^147^ ^]^ Another example of bone marrow biofabrication was demonstrated by Moore et al.^[^ ^148^ ^]^ wherein a printable bioink consisting of methylcellulose and alginate modeled the rheological and ultrastructural properties of bone marrow tissue while exhibiting high cell viability over time. Overall, employing bioprinting techniques for the fabrication of immune‐based 3D structures and chips present a promising direction of future research to better elucidate the current unknowns in the role of the immune system in the drug development process.

Apart from all its challenges, OOC technology has significant potential for various uses in the future. To ensure successful personalized drug developed, these platforms should aim to be developed with patient‐derived materials, such as patient tissue, dECM, and patient‐derived iPSCs.^[^ ^149^ ^]^ However, there are some limitations which must be acknowledged; patient‐derived tissues often require invasive collection methods, provide limited numbers of cells, and the resulting cells can often have low proliferative potentials. Instead, researchers circumvent this by using patient iPSCs to differentiate them into targeted tissue cells with which to fabricate OOCs in personalized medicine.^[^ ^150^, ^151^ ^]^ Additionally, using iPSCs derived from both healthy individuals and affected patients has allowed researchers to achieve in vitro modeling of various diseases.^[^ ^152^, ^153^, ^154^ ^]^ Moreover, another powerful hybrid drug screening tool that has shown promise is the integration of iPSC‐based organoids and OOC platforms (organoids‐on‐chip). This approach entails using iPSCs which are differentiated and self‐organized to develop organotypic microtissues and mimic real tissue microstructures and functions.^[^ ^155^, ^156^, ^157^ ^]^ Advanced models such as this could be used to model and analyze rare human diseases, as well as driving novel drug development.

As apparent in the literature, most research into OOCs is related to a single targeted organ. The primary goal of OOCs is to integrate multiple organs into a single chip to achieve human‐on‐chip for studying the synergistic effect of new drugs on more than organ at once.

## 7 Conclusion

In conclusion, OOCs are promising biomedical tools for in vitro drug screening. However, they currently still face challenges hindering their extensive clinical application, such as in achieving sufficient complexity and similarity to human tissues and organs. 3D‐printed tissues integrated with microfluidic systems have the potential to offer an alternative to animal studies. Accordingly, different strategies using 3D bioprinting for creating well‐developed biomimetic tissue models were reviewed in this paper, along with additive manufacturing applications for the fabrication of microfluidic devices. The next generation of OOCs will not only be able to take advantage of high‐quality bioprinting technology and microfluidics, but their integration with human‐derived primary cells and iPSCs will serve to advance the field of precision medicine.

## Conflict of Interest

The authors declare no conflict of interest.
